# Supplementary material for: Transcriptome Analysis to Elucidate the Effects of Milk Replacer Feeding Level on Intestinal Function and Development of Early Lambs
Source: Animals (Basel). 2023 May 24;13(11):1733. doi: 10.3390/ani13111733 (PMC10251907; doi:10.3390/ani13111733)
Supplement: Supplementary file 1 [file animals-13-01733-s001.zip › Figure S1.pdf]

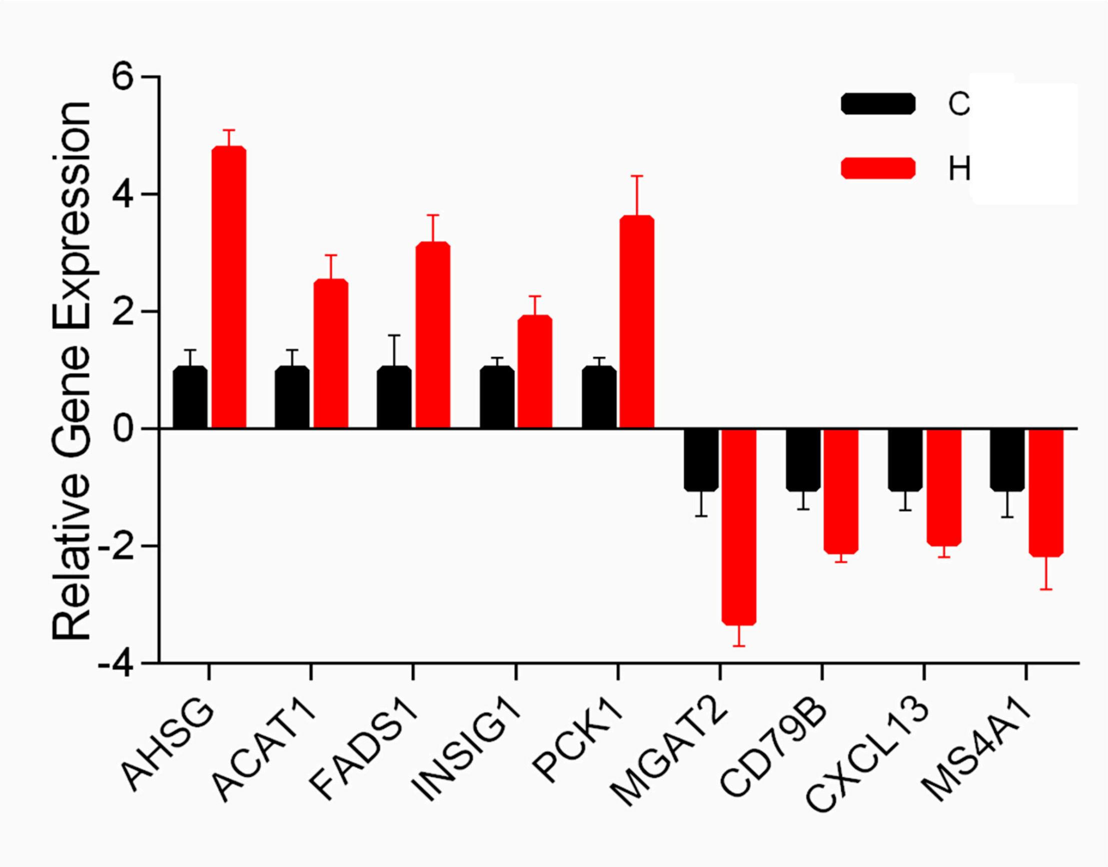

Figure S1. Validating the expression of differentially expressed genes. The relative gene expression was calculated as $2^{-\Delta\Delta}$CT, and the fold change was visualized in the figure.
