# Supplementary material for: Transcriptome Analysis to Elucidate the Effects of Milk Replacer Feeding Level on Intestinal Function and Development of Early Lambs
Source: Animals (Basel). 2023 May 24;13(11):1733. doi: 10.3390/ani13111733 (PMC10251907; doi:10.3390/ani13111733)
Supplement: Supplementary file 1 [file animals-13-01733-s001.zip › Table S1.pdf]

**Table S1.** Primers for the genes based on the *ovis* sequences obtained from the NCBI database

| Gene | Primer sequences (5'–3') | Product length | NCBI accession no. |
|---|---|---|---|
| *AHSG* | F: ATCCCGCTTGACCCGATT | 108bp | NM_001009802.1 |
| | R: CCGAGGAAGGTGCTTGTTG | | |
| *ACAT1* | F: GGGTGCAGGCTTACCTATTTCTAC | 180bp | XM_015100759.1 |
| | R: GGTGTTGCTCCTCTGTTCATTAC | | |
| *FADS1* | F: GCCCTACAACTACCAGCACAAA | 154bp | XM_004019593.3 |
| | R: GAGGAAGATGCGGACGTAGAA | | |
| *INSIG1* | F: CATCGCCACCATCTTCTCC | 129bp | XM_015095466.1 |
| | R: CACTCCCGCTTGAACTTGTG | | |
| *PCK1* | F: CGGGATGGAAAGTGGAGTG | 134bp | XM_004014441.2 |
| | R: GGGGTTTGTCTTCACAGAGGTT | | |
| *MGAT2* | F: CAAACTGCTGCTGGACTCACTT | 155bp | XM_004010499.3 |
| | R: ACTGAATGCTGAAAGGAAAGAACAC | | |
| *CD79B* | F: TCATCGTGCCCATCTTCCT | 146bp | XM_004013025.3 |
| | R: CGACCACTTCACCTCTCCTGT | | |
| *CXCL13* | F: CCATTTCCCCATTCATCTCATT | 135bp | XM_004009931.2 |
| | R: GCTTGTGTCCTTTTGGCTTG | | |
| *MS4A1* | F: AAGACTCGCCAATAGGAAACGA | 120bp | XM_004016513.3 |
| | R: CGGAAATGCGGGTGAATAAG | | |
| *β-Actin* | F: CCTGCGGCATTCACGAA | 59bp | NM_001009784.2 |
| | R: GCGGATGTCGACGTCACA | | |
