# Supplementary material for: Transcriptome Analysis to Elucidate the Effects of Milk Replacer Feeding Level on Intestinal Function and Development of Early Lambs
Source: Animals (Basel). 2023 May 24;13(11):1733. doi: 10.3390/ani13111733 (PMC10251907; doi:10.3390/ani13111733)
Supplement: Supplementary file 1 [file animals-13-01733-s001.zip › Table S2.pdf]

Table S2. The sequencing data mapping statistics and reference genome comparison

Items	Group		SEM
	C	H	
Valid reads	48375098.57	45209943.14	1071083.06
Mapped reads（%）	88.93	89.77	0.27
Unique mapped reads（%）	61.1	60.54	0.22
Multi mapped reads（%）	27.83	29.23	0.35
PE mapped reads（%）	82.72	84.14	0.45

Note: C: control group, H: intensive MR feeding group.
